# Multi-Centre Evaluation of the Determine HIV Combo Assay when Used for Point of Care Testing in a High Risk Clinic-Based Population

**DOI:** 10.1371/journal.pone.0094062

**Published:** 2014-04-08

**Authors:** Damian P. Conway, Martin Holt, Anna McNulty, Deborah L. Couldwell, Don E. Smith, Stephen C. Davies, Philip Cunningham, Phillip Keen, Rebecca Guy, on behalf of the Sydney Rapid HIV Test Study

**Affiliations:** 1 The Kirby Institute, University of New South Wales, Sydney, New South Wales, Australia; 2 Centre for Social Research in Health, University of New South Wales, Sydney, New South Wales, Australia; 3 Sydney Sexual Health Centre, Sydney Hospital, Sydney, New South Wales, Australia; 4 School of Public Health and Community Medicine, University of New South Wales, Sydney, New South Wales, Australia; 5 Western Sydney Sexual Health Centre, Western Sydney Local Health District, Sydney, New South Wales, Australia; 6 Sydney Emerging Infections and Biosecurity Institute, University of Sydney, Sydney, New South Wales, Australia; 7 Albion Centre, Surry Hills, Sydney, New South Wales, Australia; 8 North Shore Sexual Health Service, Royal North Shore Hospital, St Leonards, Sydney, New South Wales, Australia; 9 St Vincent's Centre for Applied Medical Research, University of New South Wales, Sydney, New South Wales, Australia; 10 NSW State Reference Laboratory for HIV, St Vincent's Hospital, Darlinghurst, Sydney, New South Wales, Australia; New York University, United States of America

## Abstract

**Background:**

Determine HIV Combo (DHC) is the first point of care assay designed to increase sensitivity in early infection by detecting both HIV antibody and antigen. We conducted a large multi-centre evaluation of DHC performance in Sydney sexual health clinics.

**Methods:**

We compared DHC performance (overall, by test component and in early infection) with conventional laboratory HIV serology (fourth generation screening immunoassay, supplementary HIV antibody, p24 antigen and Western blot tests) when testing gay and bisexual men attending four clinic sites. Early infection was defined as either acute or recent HIV infection acquired within the last six months.

**Results:**

Of 3,190 evaluation specimens, 39 were confirmed as HIV-positive (12 with early infection) and 3,133 were HIV-negative by reference testing. DHC sensitivity was 87.2% overall and 94.4% and 0% for the antibody and antigen components, respectively. Sensitivity in early infection was 66.7% (all DHC antibody reactive) and the DHC antigen component detected none of nine HIV p24 antigen positive specimens. Median HIV RNA was higher in false negative than true positive cases (238,025 vs. 37,591 copies/ml; p = 0.022). Specificity overall was 99.4% with the antigen component contributing to 33% of false positives.

**Conclusions:**

The DHC antibody component detected two thirds of those with early infection, while the DHC antigen component did not enhance performance during point of care HIV testing in a high risk clinic-based population.

## Introduction

People with acute HIV infection contribute disproportionately to HIV transmissions due to their high viral loads [1]–[2]. Mathematical modelling and phylogenetic analysis estimate that those with acute infection account for 19–50% of sexual HIV transmissions in a range of populations and settings [3]–[5]. Cohort study data show that risk of HIV transmission correlates with viral load [6] and is higher during acute and early infection compared with established infection [7]–[8]. Earlier identification of HIV infection and initiation of treatment may have both individual [9]–[10] and public health [11]–[12] benefits.

While automated fourth generation HIV immunoassays [13] and pooled HIV nucleic acid testing [14] have enabled identification of those with acute infection prior to development of HIV-specific antibodies, these methods are resource intensive and unsuitable for testing outside laboratories. Rapid HIV testing has expanded access to testing in resource poor settings with limited laboratory infrastructure [15] and in high risk or hard to reach populations in resource rich settings [16]. However, if HIV antibody only rapid tests are the mainstay of testing in these settings the longer window periods of such assays may mean many acute HIV infections are missed, especially in high incidence populations [17]–[18]. The Determine HIV Combo (DHC) has been approved for use by regulatory authorities in Europe, Australia and the United States (US) and is the first point of care assay containing both HIV antibody and antigen components specifically designed to increase sensitivity in patients recently infected with HIV.

The manufacturer package insert [19] and an initial laboratory-based evaluation [20] indicated DHC had the capacity to detect acute HIV infections. However, subsequent studies reported that DHC performance varied by whether serum or fingerstick blood specimens were used [21] and was less favourable during field evaluation [22]. Though laboratory studies enable performance evaluation using a range of accessible and characterised samples, including seroconversion panels, clinic-based or field studies involving freshly collected specimens from the target population in which the test will be used are necessary to adequately evaluate point of care assay performance [23]–[24].

In order to gain a better understanding of the potential of DHC for use as a point of care screening assay, we assessed its performance when used by sexual health clinicians for HIV testing in a high risk population of gay, bisexual and other men who have sex with men (MSM).

## Methods

### Setting

The study was conducted in four free access publicly funded sexual health clinics with high caseloads of MSM: two in central (Sydney Sexual Health Centre and Albion Centre) and two in suburban Sydney (Western Sydney Sexual Health Centre and North Shore Sexual Health Service). Among MSM surveyed in New South Wales (NSW) in 2013, 45% of men who had ever tested reported their last HIV test was at a public sexual health clinic [25]. In Australia, 85% of new HIV diagnoses are in MSM [26], HIV prevalence among MSM in large cities is around 12% [27] and HIV incidence in MSM is 1–2% [28]–[29].

### Ethical statement

The study was approved by the Human Research Ethics Committees of St Vincent's Hospital, Sydney and UNSW Australia (The University of New South Wales). Written informed consent was obtained from all patient participants.

### Study design

During this 20 month (October 2011 to July 2013) cross-sectional prospective study, clinicians offered rapid testing with DHC to MSM 18 years or older presenting for HIV testing. DHC results were referenced to conventional HIV serology conducted in parallel for all patients in the study (details below). Men could participate more than once.

### Staff roles and training

At the four participating clinics, doctors and nurses offered DHC to patients, performing and reading the test, with counsellors available to support patients with positive test results. In all, 68 clinic staff attended training provided by staff from the Kirby Institute, NSW State Reference Laboratory for HIV and National Serology Reference Laboratory. Training covered policy, theoretical and practical aspects of rapid HIV testing, including quality assurance, testing with DHC, and interpreting results.

### Testing procedures

Clinicians obtained informed consent and collected venipuncture specimens for conventional testing and fingerstick blood specimens for rapid HIV testing, with the latter applied to the DHC assay in accordance with the manufacturer's instructions [19]. After 20 minutes, DHC results were scored by clinic staff as non-reactive (patient's specimen did not react with test lines), reactive (patient's specimen reacted with one or both test lines) or invalid (control line absent). Patients received DHC results during their clinic visit, with those receiving reactive results offered counselling and support. Patients with false negative DHC results were recalled to clinics to receive their conventional results and appropriate care. Follow up HIV testing was recommended for patients with false positive results. In the first ten months, clinicians also asked men to complete surveys on their demographics, risk behaviour, and previous HIV testing history.

### Quality management

DHC shipments from the manufacturer (Alere Pty Ltd, Sinnamon Park, Queensland, Australia) were batch tested at NSW State Reference Laboratory for HIV prior to release for patient testing after demonstrating expected reactivity with known HIV positive and negative serum control specimens. National Serology Reference Laboratory provided the serum control testing specimens used by site staff at weekly intervals.

### Reference laboratory assays

DHC results were classified as true or false with reference to results of the laboratory assays which are standard of care in this setting: fourth generation HIV screening immunoassay (Architect HIV Ag/Ab Combo, Abbott Diagnostics, Wiesbaden, Germany; or Elecsys HIV Combi PT, Roche Diagnostics, Mannheim, Germany), supplementary HIV antibody (Serodia HIV-1 Antibody particle agglutination assay, Fujirebio, Tokyo, Japan; or Genscreen HIV 1&2 Antibody EIA, Bio-Rad Laboratories, Redmond, WA), HIV p24 antigen immunoassay (Genscreen HIV-1 p24 Antigen, Bio-Rad Laboratories, Redmond, WA) and HIV Western blot (HIV Western Blot 2.2, MP Diagnostics, Singapore). In men diagnosed HIV-positive, HIV RNA (HIV Cobas Taqman 48 v2.0, Roche Diagnostics, Branchburg, NJ), CD4 T-cell count (Canto II flow cytometer, Becton Dickenson, San Jose, CA) and genotype (Vircotype HIV-1, Janssen Diagnostics, Beerse, Belgium) tests were performed.

### Testing algorithm and definitions

If the fourth generation laboratory screening HIV immunoassay was negative, the patient's true HIV status was deemed negative. Supplementary HIV antibody, HIV p24 antigen and Western blot testing were performed on all specimens positive by the fourth generation HIV screening immunoassay with true HIV status deemed positive if consistent with the national case definition [30]. HIV cases were classified as early, acute or recent HIV infection according to the definitions used in the US Department of Health and Human Services Guidelines for Antiretroviral Therapy in HIV Infection. Acute infection was defined as HIV RNA or p24 antigen positive, but HIV antibody negative; recent infection was defined as HIV antibody positive, but infected within the last six months; while early infection referred to cases of acute and recent infection combined [31]. Previous or repeat samples from the same individual were identified via their clinic identification number and matched to patient notes retrieved by site staff. Previous testing history was based on testing conducted at the clinic, or self-reported by the patient. Cases with no history of testing HIV negative in the last six months were classified as recent infection if their laboratory test results were consistent with that in the Fiebig classification [32].

### Statistical analyses

Invalid results were excluded from DHC performance analyses. Sensitivity, specificity, and negative and positive predictive values were calculated with 95% confidence intervals (CIs) for DHC overall (defining test positivity as reactivity in either the antibody or the antigen component) and for the antibody and antigen components separately. Sensitivity was assessed in HIV cases classified into acute, recent and early infection subgroups.

Men testing HIV-positive were characterised by HIV RNA, p24 antigen titre, subtype, CD4 count and date of last negative HIV test. Behavioural characteristics in HIV-positive and HIV-negative men and clinical status (HIV RNA, CD4 count) in men with true positive and false negative rapid tests were compared using Pearson chi square, Fisher's exact and Mann-Whitney tests. Probability values of less than 0.05 were considered significant and analyses were performed using Stata (Release 12, StataCorp LP, College Station, Texas).

## Results

### Characteristics of men overall and according to HIV infection status

During the 20-month study period, 2468 men at four sites had 3195 DHC tests plus conventional HIV serology performed (breakdown by site in table S1 in file S1). Reference tests confirmed new HIV diagnoses in 39 of 2468 men with no significant difference in median age between HIV-positive and HIV-negative men (30 vs 31 years; p = 0.404).

Of survey participants, 89.9% of men reported previous HIV testing and, in the last six months, 28.4% reported more than ten male sexual partners and 35.0% unprotected anal intercourse with casual male partners (table S2 in file S1). Compared with HIV-negative men, HIV-positive men were more likely to report unprotected anal intercourse with casual male partners in the last six months (57.1% vs 34.6%; p = 0.032).

Among 39 HIV-infected men, 86% had HIV subtype B (the remainder having subtype C or recombinant forms), median CD4 count was 440 (range 10–950) cells/mm^3^ and median HIV RNA was 39859 (range 1251–3898751) copies/ml (table 1).

**Table 1:** Laboratory characteristics of men diagnosed as HIV-positive.

Characteristic	True positive (n = 34)* †	False negative (n = 5)* †	All combined (n = 39)†	Test & p-value
HIV subtype B (N; %)	28 (85)	3 (100)	31 (86)	χ^2^ = 0.53; 0.630
Median CD4 count (range; cells/mm^3^)	410 (10–950)	495 (380–920)	440 (10–950)	z = 0.88; 0.379
Median HIV RNA (range; copies/ml)	37591 (1251–1096819)	238025 (93197–3898751)	39859 (1251–3898751)	z = 2.30; 0.022
Median p24 antigen titre (range; pg/ml)	114 (66–366)	166 (86–701)	115 (66–701)	z = 0.98; 0.325
Early HIV infection (N; %)	8 (24)	4 (80)	12 (31)	χ^2^ = 6.53; 0.025

*Classification of rapid test result is that for the test overall;

† Men with missing data excluded; mm^3^ = cubic millimetre; ml = millilitre; pg = picograms; χ^2^ = Fisher's exact test; z = Mann-Whitney test.

### Classification and characteristics of HIV cases with early infection

Of 39 specimens HIV-positive by reference tests, 12 (30.7%) met the criteria for early infection, of which three were classified as acute infections and nine as recent infections (table 2). There were three p24 antigen positive cases (not shown) which were reactive by the DHC antibody component and all laboratory assays, but did not meet the criteria for early infection as their last negative HIV test was 12–24 months ago. These cases had p24 antigen titres of 66, 114 and 366 pg/ml and HIV RNA of 27271, 432141 and 1096819 copies/ml, respectively. The only DHC false negative case not classified as having early infection was positive on laboratory fourth generation and Western blot assays and p24 antigen negative in a patient who last tested HIV-negative 12 months previously.

**Table 2:** Characteristics of cases of early HIV infection.

Group	Case	Rapid test result		Last negative HIV test (months)	4^th^ Gen HIV Ag/Ab	Supp HIV Ab	Western blot	HIV p24 Ag titre (pg/ml)	HIV RNA (copies/ml)	CD4 count (cells/mm^3^)	HIV subtype
		Antibody	Antigen								
Acute	1	NR	NR	11	POS	NEG	NEG	701	3898751	510	B
	2	NR	NR	9	POS	NEG	NEG	86	93197	480	NA
	3	NR	NR	4	POS	NEG	NEG	115	99171	380	B
Recent	4	NR	NR	65	POS	WK POS	IND	217	376879	920	B
	5	R	NR	8	POS	POS	IND	66	568763	840	C
	6	R	NR	6	POS	POS	IND	143	274918	440	B
	7	R	NR	3	POS	POS	IND	NEG	13003	870	B
	8	R	NR	5	POS	POS	IND	NEG	23843	580	B
	9	R	NR	2	POS	POS	POS	NEG	8422	260	CRF02_AG
	10	R	NR	3	POS	POS	POS	NEG	37591	600	B
	11	R	NR	4	POS	POS	POS	NEG	102326	510	B
	12	R	NR	6	POS	POS	POS	NEG	29500	515	B

Acute = HIV RNA or p24 antigen positive, but HIV antibody negative; Ag = antigen; Ab = antibody; IND = indeterminate; ml = millilitre; mm^3^ = cubic millimetre; NA = not available;

NEG = negative; NR = non-reactive; R = reactive; recent = HIV antibody positive, but infected within last 6 months; pg = picograms; POS = positive; WK POS = weak positive.

CD4 count was higher in men with, compared to men without, early infection (513 vs 380 cells/mm^3^; p = 0.030), whereas there was no significant difference in median HIV RNA (96184 vs 39012 copies/ml; p = 0.153).

### Performance of the DHC assay overall

**Figure 1:**
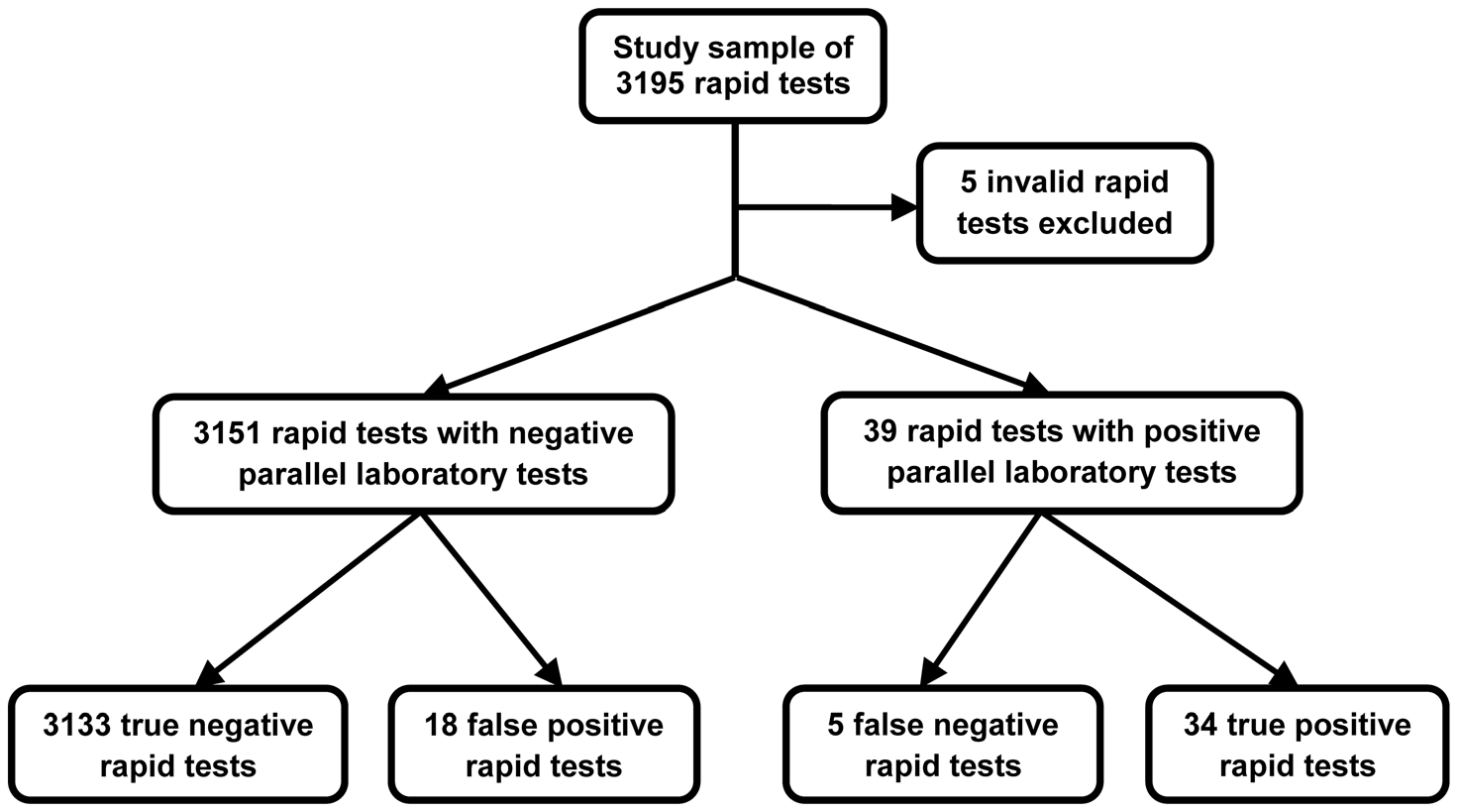
Flowchart of parallel rapid and laboratory testing with classification of DHC results (overall).

Of 3195 specimens tested, five (0.2%) with invalid DHC results were excluded (figure 1). Of the remainder, 39 were HIV-positive by reference tests (1.2%), of which 34 were DHC reactive (overall sensitivity = 87.2%; 95%CI 71.8–95.2) and five were DHC false negative (table 3). Of 3151 specimens negative by reference testing, 3133 were DHC non-reactive (overall specificity = 99.4%; 95%CI 99.1–99.7) and 18 were DHC false positive. Of the 18 patients with false positive DHC results, 14 had follow up testing where they tested HIV negative. The remaining four patients declined or did not attend for further testing. Eight false positives occurred in the first three months of the study, compared with 1–3 false positives per quarter thereafter. Of 52 DHC reactive tests, 34 were true positive by reference testing (positive predictive value = 65.4%; 95%CI 50.8–77.7) (table 3). There were no significant differences between DHC true positive versus false negative cases in terms of median CD4 count (410 vs 495 cells/mm^3^; p = 0.379); whereas, median HIV RNA was higher in DHC false negative cases (37591 vs 238025 copies/ml; p = 0.022) (table 1).

**Table 3:** Performance of Determine HIV Combo compared with laboratory serology*.

Characteristic	Antigen (n = 3190)	Antibody (n = 3190)	Overall (n = 3190)
True positive	0	34	34
False negative	9	2	5
True negative	3175	3140	3133
False positive	6	14	18
Sensitivity (%; 95% CI)	0.00 (0.00–37.12)	94.44 (79.99–99.03)	87.18 (71.77–95.18)
Specificity (%; 95% CI)	99.81 (99.57–99.92)	99.56 (99.24–99.75)	99.43 (99.08–99.65)
Negative predictive value (%; 95% CI)	99.72 (99.44–99.86)	99.94 (99.74–99.99)	99.84 (99.61–99.94)
Positive predictive value (%; 95% CI)	0.00 (0.00–48.32)	70.83 (55.74–82.60)	65.39 (50.84–77.67)

*Fourth generation immunoassay, supplementary HIV antibody, p24 antigen and Western blot tests; CI = confidence interval.

### Performance of the DHC antibody & antigen components

Of 3190 specimens, 36 were HIV antibody positive by laboratory tests (three of 39 confirmed HIV cases were positive by fourth generation HIV immunoassay, but negative on supplementary HIV antibody and HIV Western blot testing [table 2]). Of 36 HIV antibody positive cases, 34 were DHC antibody reactive (sensitivity = 94.4%; 95%CI 80.0–99.0) and two were false negatives (table 3). Of 31 specimens that were both HIV antibody and Western blot positive by reference testing, 30 were DHC antibody reactive (sensitivity = 96.8%; 95%CI 81.5–99.8).

Nine of 3190 specimens were HIV p24 antigen positive by reference testing, of which zero were DHC antigen reactive (sensitivity = 0.0%; 95%CI 0.0–37.1) (table 3). The p24 antigen titres of the nine specimens which were DHC antigen false negative ranged from 66–701 (median 115) pg/ml (table 1). Of a total of 18 false positive DHC results, four were antigen reactive, 12 antibody reactive and two both antigen and antibody reactive. Hence, the antigen component contributed to 6 of 18 (33.3%) false positive results.

### Sensitivity of the DHC assay in acute, recent and early infection

None of the three specimens classified as acute were DHC reactive, whereas eight of nine specimens classified as recent were DHC antibody reactive and one was false negative (table 2). Thus, eight of 12 specimens with early infection were DHC antibody reactive (sensitivity = 66.7%; 95%CI 35.4–88.7) and four were false negative. Median HIV RNA in the four DHC false negative specimens with early infection was 238025 (range 93197–3898751) copies/ml (table 1). Median p24 antigen titre was 129 (range 66–701) pg/ml in the six specimens with early infection that were antigen positive on reference testing, none of which were DHC antigen reactive.

## Discussion

This is the largest clinical evaluation of DHC reported to date; with sensitivity stratified by early infection and performance referenced to parallel laboratory testing, including a fourth generation screening immunoassay. We have assessed operational performance of DHC when used by clinicians (as opposed to laboratory technicians) in a high risk clinic-based population of gay and bisexual men. Among 39 men diagnosed as HIV-positive (12 with early infections), DHC sensitivity was 87.2% overall and 94.4% and 0% for the antibody and antigen components, respectively. The sensitivity in early infection of 66.7% was less than the 92% reported in the package insert [19] and none of the nine HIV p24 antigen positive specimens were detected by the DHC antigen component. Specificity of the DHC antibody (99.6%) and antigen (99.8%) components was consistent with the package insert, with the antigen component contributing to 6 of 18 (33%) of false positive results.

There have been 16 evaluations of DHC performance previously published in the literature, but only five have used freshly collected fingerstick blood samples from a clinic or community-based population [21]–[22], [33]–[35]. However, of these: one utilised both clinical and technical staff to perform rapid testing [21]; one performed laboratory reference testing only for reactive rapid tests (thus sensitivity and specificity were not assessed) [34]; one performed testing in known HIV-positive patients only [33] and another did not inform patients of the DHC result (which may have resulted in observer bias in interpreting results) [35]. Other limitations of published evaluations include: not reporting who performed rapid testing [33], [35]–[39] or which population was tested [20], [37], [40]; and not fully characterising HIV cases in terms of HIV RNA, p24 antigen titre and subtype data [18], [20], [33]–[36], [39]–[41].

DHC was designed to increase sensitivity of rapid HIV testing in patients with early infection, whereas we have shown the DHC antigen component contributed to 33% of false positives and did not provide any performance advantage in early infection when used with fingerstick blood specimens in a high risk population. Three other clinic studies using fingerstick blood specimens also found that the DHC antigen component failed to detect specimens from patients with acute infection [21]–[22], [35], whereas another found that DHC detected 3 of 5 (60%) patients with acute infection [33].

In laboratory studies using serum or plasma specimens, sensitivity of the DHC antigen component in patients with acute infection varies considerably, from as low as 2% [42] to as high as 87% [20]. The DHC package insert reports 25 pg/ml as the lower limit of p24 antigen detection and independent studies confirmed the lower limit to be 20–50 pg/ml when using serial dilutions of p24 antigen serum control [37], [42]. However, the p24 antigen lower limit of detection for clinical specimens appears higher: in one study 51 antigen positive plasma specimens ranging 8–457 (median 94) pg/ml remained undetected [42]; and in our study nine fingerstick blood specimens ranging 66–701 (median 115) pg/ml remained undetected. Hence, the DHC antigen lower limit of detection when used with clinical specimens, as opposed to serum control specimens, requires further investigation and clarification. Previous research also suggests the DHC antigen component is likely to be non-reactive in specimens both HIV antibody and p24 antigen positive [40], [42], possibly due to lack of free (unbound) antigen [22]. The relative insensitivity of the DHC antigen component could possibly be related to p24 epitope coverage in the cocktail of detection antibodies included in that component. Antigen detection in conventional immunoassays involves optimum conditions including pH buffered isotonic diluents, ideal temperature and prolonged incubation periods to obtain antigen-antibody complex formation, whereas rapid HIV testing does not provide such ideal binding conditions and this may result in loss of sensitivity.

In contrast to antigen performance, in our evaluation the DHC antibody component detected two thirds of those with early infection and 94% of those confirmed as antibody positive by laboratory testing. Laboratory studies using serum or plasma specimens have found DHC antibody sensitivity in patients with known or established infection to be 100% [20]–[21], [36], [41]–[42]; whereas DHC antibody sensitivity in clinic or field studies using fingerstick blood has ranged from 95% [35] to 99% [22].

Other factors that could potentially influence DHC performance include HIV RNA and subtype. In the laboratory, the DHC antigen component has detected five of 17 serum specimens from patients with acute infection (29%), four of whom had HIV RNA exceeding 10 million copies/ml [38]. In our study, median HIV RNA was higher in those with false negatives than true positives (238025 vs 37591 copies/ml), reflecting their earlier stage of HIV infection, but patients with these higher viral loads remained undetected. Regarding subtype, DHC antibody component sensitivity of 100% has been reported in a range of serum and plasma HIV-1 group O and M variant specimens [20] while another study using fingerstick blood reported sensitivity of 84% in non-B subtypes [21]. In our study, 14% of HIV-infected men had non-B HIV subtypes, but as all non-B subtype specimens were detected this did not appear to influence DHC sensitivity.

Though DHC specificity was high in our evaluation, false positives generate stress and anxiety with 18 patients in our study having to wait for laboratory test results to clarify their HIV status. Though our clinician staff were appropriately trained, they had to develop experience in administering point of care HIV testing. While there are patient (interfering antibodies), device (batch to batch variation) and operator (experience and skill) factors involved in false positivity, the excess of false positives in the first three months of our study may have been related to staff inexperience. Operator experience has been shown to affect interpretation of results and performance of point of care HIV assays [43]–[44]. Though there were four patients with false positive DHC tests who did not attend for follow up testing, we do not believe these patients could have had acute infections not detected by the Abbott Architect screening immunoassay used for parallel laboratory testing in these cases. DHC has repeatedly been shown to be less sensitive than the Abbott Architect assay in independent studies using serum and plasma specimens from patients with acute HIV infection [18], [38], [45], [46] and studies using fingerstick blood specimens from patients known to have HIV infection [21] and undergoing clinic based screening [35].

The main strengths of our study in terms of real world evaluation were: being clinic-based; using fingerstick blood specimens; the large sample size; using a large number of trained staff in testing across four sites; and reporting an extended range of laboratory characteristics for patients diagnosed HIV-positive.

Our evaluation also had some potential limitations. Firstly, our findings among clinic-based MSM undergoing point of care DHC testing with fingerstick blood specimens may not be generalisable outside that population, setting and specimen type. Secondly, the standard of care fourth generation laboratory screening immunoassays used in our local setting are more sensitive in early infection than second and third generation laboratory HIV assays used in other jurisdictions. Hence, in settings where these less sensitive laboratory assays are used DHC performance may be comparable to or better than the standard of care. Thirdly, DHC testing was typically performed and interpreted (with a second reader) by the clinician consulting with the patient being tested. It is possible that clinicians who are not blinded to the HIV risk assessment for the patient may be biased in their interpretation of DHC results. However, in a busy clinical setting it may be impractical to roster test readers who are blinded to patient risk assessment. Finally, some investigators define recent infection on the basis of non-reactivity to detuned immunoassay testing. The latter was not part of our evaluation, but we believe defining recent infection by HIV antibody positivity in patients infected within the last six months is valid and would be useful to clinicians implementing rapid HIV testing.

The approval of the DHC assay by regulatory authorities in Europe, Australia and the US highlights the need for both clinicians and patients to be well informed regarding the limitations of this assay (and point of care rapid HIV testing generally) in detecting cases of early HIV infection. Some authors support using a fourth generation rapid HIV test such as DHC to detect acute HIV infection in clinical settings [45] and others have proposed that antibody only rapid HIV tests should be supplemented with p24 antigen or nucleic acid testing for screening high incidence MSM populations [47]. In laboratory studies using plasma specimens from patients with acute HIV infection, DHC has demonstrated sensitivity that is higher than other rapid HIV tests and in between third and fourth generation laboratory HIV immunoassays [18], [45], [46]. However, this does not reflect typical point of care use of the assay and the sensitivity in acute infection provided by the antigen component in some studies [20], [45], [46] has not been shown in others [22], [36], [42]. The performance of DHC reported in various settings (laboratory versus point of care) and specimen types (serum, plasma and fingerstick whole blood) should be considered when making decisions regarding the use of DHC for screening in high incidence populations.

In conclusion, the DHC antigen component failed to detect p24 antigen in any confirmed HIV p24 antigen positive specimen collected in our study, indicating that the antigen component does not perform well with fingerstick blood specimens collected at the point of care. In contrast, the DHC antibody component detected two thirds of those with early infection and 94% of all confirmed HIV-positive cases. Given the public health implications of a false negative result in a patient with acute HIV infection and high HIV RNA, a robust point of care assay that reliably detects acute infection is urgently needed. The false negative results in our study and other studies also demonstrate that where point of care rapid HIV testing is being used, patients with risk factors for recent infection should be identified so that conventional HIV serology or nucleic acid testing can be performed. Finally, our study demonstrates the importance of point of care test evaluations which include the intended testers and target population and involve collection of fresh specimens, reflecting typical use. Data from laboratory-based evaluations should not be expected to predict performance when rapid tests are used with fingerstick blood specimens by clinicians at the point of care.

## Supporting Information

File S1 **Table S1 & Table S2.** Table S1 Testing data & trained staff by study site.Table S2 Patient behavioural characteristics.
(PDF)
