# Correction: Multi-Centre Evaluation of the Determine HIV Combo Assay when Used for Point of Care Testing in a High Risk Clinic-Based Population

**DOI:** 10.1371/journal.pone.0103399

**Published:** 2014-07-16

**Authors:** The PLOS ONE Staff

In the Results subsection of the Abstract, there is an error in the first sentence. Please see the corrected sentence below:

“Of 3190 evaluation specimens, 39 were confirmed as HIV-positive (12 with early infection) and 3151 were HIV-negative by reference testing.”
