# Supplementary material for: Multi-Centre Evaluation of the Determine HIV Combo Assay when Used for Point of Care Testing in a High Risk Clinic-Based Population
Source: PLoS One. 2014 Apr 8;9(4):e94062. doi: 10.1371/journal.pone.0094062 (PMC3979750; doi:10.1371/journal.pone.0094062)
Supplement: File S1 — Table S1 & Table S2. Table S1 Testing data & trained staff by study site.Table S2 Patient behavioural characteristics. (PDF) [file pone.0094062.s001.pdf]

Conway DP, Holt M, McNulty A, Couldwell DL, Smith DE, Davies SC, Cunningham P, Keen P, Guy R on behalf of the Sydney Rapid HIV Test Study. Multi-centre evaluation of the Determine HIV Combo assay when used for point of care testing in a high risk clinic-based population. PLOS One 2014.

Supporting Information File S1

Table S1 Testing data & trained staff by study site

Table S2 Patient behavioural characteristics

Table S1 Testing data & trained staff by study site

Study site	Sydney SHC	Albion Centre	Western Sydney SHC	North Shore SHS
Patient participants (N, %)	1565 (63.4)	285 (11.6)	473 (19.2)	145 (5.9)
Rapid tests performed (N, %)	2025 (63.4)	348 (10.9)	653 (20.4)	169 (5.3)
True negative results* (N)	1986	332	647	168
False negative results* (N)	3	2	0	0
True positive results* (N)	22	9	3	0
False positive results* (N)	11	4	3	0
Invalid rapid tests (N)	3	1	0	1
4th generation laboratory screening assay	Abbott Architect	Abbott Architect	Abbott Architect	Roche Elecsys Combi PT
Clinician staff trained (N, %)	24 (35.3)	12 (17.7)	20 (29.4)	12 (17.7)

*Refers to rapid test performance overall; SHC=sexual health centre; SHS=sexual health service

Table S2 Patient behavioural characteristics*

Characteristic	HIV-positive (n=21)†	HIV-negative (n=1072)†	Total (n=1093)†	Chi-square; p-value
Identifies as gay	19 (95.0%)	937 (87.7%)	956 (87.9%)	‡0.97; 0.280
Ever tested for HIV	20 (95.2%)	961 (89.8%)	981 (89.9%)	‡0.67; 0.357
Tests twice a year or more often	9 (42.9%)	584 (54.8%)	593 (54.6%)	1.18; 0.277
More than 10 sexual partners in last 6 months	8 (38.1%)	299 (28.2%)	307 (28.4%)	1.00; 0.318
UAI with casual partners in last 6 months	12 (57.1%)	367 (34.6%)	379 (35.0%)	4.62; 0.032
UAI with regular partners in last 6 months	11 (52.4%)	604 (57.1%)	615 (57.0%)	0.19; 0.666

*Data from men participating in a survey conducted in the first 10 months of the study only

†Men with missing data excluded; ‡Fisher's exact test; UAI=unprotected anal intercourse
